# Imputation of DNA Methylation Levels in the Brain Implicates a Risk Factor for Parkinson’s Disease

**DOI:** 10.1534/genetics.115.185967

**Published:** 2016-07-26

**Authors:** Konrad Rawlik, Amy Rowlatt, Albert Tenesa

**Affiliations:** * Roslin Institute, University of Edinburgh, Edinburgh EH25 9RG, United Kingdom; † Medical Research Council Human Genetics Unit at the Medical Research Council Institute of Genetics and Molecular Medicine, University of Edinburgh, Edinburgh EH4 2XU, United Kingdom

**Keywords:** DNA methylation, imputation, Parkinson’s disease

## Abstract

Understanding how genetic variation affects intermediate phenotypes, like DNA methylation or gene expression, and how these in turn vary with complex human disease provides valuable insight into disease etiology. However, intermediate phenotypes are typically tissue and developmental stage specific, making relevant phenotypes difficult to assay. Assembling large case–control cohorts, necessary to achieve sufficient statistical power to assess associations between complex traits and relevant intermediate phenotypes, has therefore remained challenging. Imputation of such intermediate phenotypes represents a practical alternative in this context. We used a mixed linear model to impute DNA methylation (DNAm) levels of four brain tissues at up to 1826 methylome-wide sites in 6259 patients with Parkinson’s disease and 9452 controls from across five genome-wide association studies (GWAS). Six sites, in two regions, were found to associate with Parkinson’s disease for at least one tissue. While a majority of identified sites were within an established risk region for Parkinson’s disease, suggesting a role of DNAm in mediating previously observed genetic effects at this locus, we also identify an association with four CpG sites in chromosome 16p11.2. Direct measures of DNAm in the substantia nigra of 39 cases and 13 control samples were used to independently replicate these four associations. Only the association at cg10917602 replicated with a concordant direction of effect (*P* = 0.02). cg10917602 is 87 kb away from the closest reported GWAS hit. The employed imputation methodology implies that variation of DNAm levels at cg10917602 is predictive for Parkinson’s disease risk, suggesting a possible causal role for methylation at this locus. More generally this study demonstrates the feasibility of identifying predictive epigenetic markers of disease risk from readily available data sets.

GENOME-WIDE association studies (GWAS) have been successful in identifying associations between common genetic variations, typically SNPs, and a common complex disease or trait. However, despite this success GWAS have provided only limited information about the mechanistic role of genetic variation in the etiology of disease. In part, this result follows from the challenge to map the exact location of the causative variant and identify its functional consequence as well as the highly polygenic nature of many phenotypes targeted by GWAS. Understanding how heritable tissue-specific cellular phenotypes (that is, intermediate phenotypes) vary with respect to both the genotype and complex disease assists us in the construction of medically relevant biological networks. To this end there has been an increasing interest in both GWAS of intermediate phenotypes (Gibbs *et al.* 2010; Zhang *et al.* 2010; Bell *et al.* 2011) and studies that link intermediate phenotypes to complex diseases or traits measured at the organism level, which we refer to as ultimate phenotypes (Emilsson *et al.* 2008; Rakyan *et al.* 2011).

Compared to traditional GWAS these studies face several challenges if the results are intended to inform on the biological mechanisms that lead to an ultimate phenotype. First, intermediate phenotypes are typically tissue and developmental stage specific (Holiday and Pugh 1975; Eckhardt *et al.* 2006; Ladd-Acosta *et al.* 2007), necessitating prior knowledge of a candidate tissue in which the intermediate phenotype mediates disease. Second, although current technology provides a means to assay intermediate phenotypes on a genome-wide scale, samples from many tissues of interest, such as human brain, are difficult to obtain. As a consequence many studies seeking to use samples from relevant tissues are of limited size with larger-scale studies limited to intermediate phenotypes observed in easily accessible tissues, most commonly whole blood samples. The imputation of difficult to assay intermediate phenotypes from genetic information, analogous to the imputation of genotypes based on reference populations, addresses these problems. We have previously employed such an imputation approach for DNA methylation (DNAm) and gene expression restricted to a small region to fine map an observed genetic association (Rowe *et al.* 2013). More recently a similar approach to that of Rowe *et al.* (2013) was followed for imputation of whole blood gene expression levels into a large GWAS cohort for various traits (Gamazon *et al.* 2015). Here we combine existing Parkinson’s disease case–control data sets with methylome-wide imputation of brain tissue-specific DNAm levels to investigate the role of this intermediate phenotype in Parkinson’s disease etiology.

The viability of DNAm imputation is not immediately apparent. However, DNAm and other intermediate phenotypes, like gene expression, do exhibit several important characteristics that distinguish them from the ultimate traits that are typically the targets of GWAS, like Parkinson’s disease. Intermediate phenotypes can exhibit comparatively large genetic effects (Dermitzakis 2008) because the number of molecular interactions separating them from genetic variants is limited, thus limiting sources of noise. Furthermore, significant portions of the heritability have been found to be regional; for example, genetic markers in a genomic region surrounding the gene or CpG locus for which expression or DNAm is measured usually explain a large portion of the phenotypic variance (Gibbs *et al.* 2010; Price *et al.* 2011; Quon *et al.* 2013). We demonstrate that, in line with the results obtained for gene expression (Gamazon *et al.* 2015), imputation based on the local regional genetic variation is feasible for a large number of DNAm sites represented by commercial arrays. This is possible because the number of marker effects to be estimated is low, which facilitates good prediction even with a moderate size of reference sample.

Association testing of imputed intermediate phenotypes with an ultimate phenotype is conceptually similar, but significantly different in motivation, to the use of alternative approaches based on multimarker association tests, like, *e.g.*, polygenic risk scores or regional heritability estimates. Polygenic risk scores are primarily a statistical tool for increasing power in association testing within a GWAS, for meta-analyses across multiple GWAS data sets, and for genomic prediction. Regional heritability estimates are similarly a tool to increase power in association testing by combining information across multiple genetic variants and reducing the multiple-testing burden. Our motivation, on the other hand, is to make use of disparate data sets and leverage their individual properties so that the large and regional effects attributable to intermediate phenotypes measured in one data set can be combined with the GWAS data for an ultimate phenotype. This allows us to gain insight into the involvement of intermediate phenotypes in the etiology of an ultimate phenotype beyond what can be discerned from each data set individually. However, such predicted associations need to be confirmed based on direct measurements. We therefore assayed DNAm in substantia nigra tissue from cases with Parkinson’s disease and controls to test whether putative associations identified using the imputation approach replicated in the target tissue.

Imputing tissue-specific DNAm for four brain regions into 15,711 individuals from five case–control GWAS, we identified six candidate DNAm sites across two regions in chromosomes 16 and 17 showing association with Parkinson’s disease case–control status. We were able to test four of these six candidate sites that passed quality control (QC) in a separate data set of 39 cases and 13 controls. One of these associations, cg10917602 in 16p11.2, replicated (*P* = 0.02) with a concordant effect direction in this data set.

## Materials and Methods

**Figure 1:**
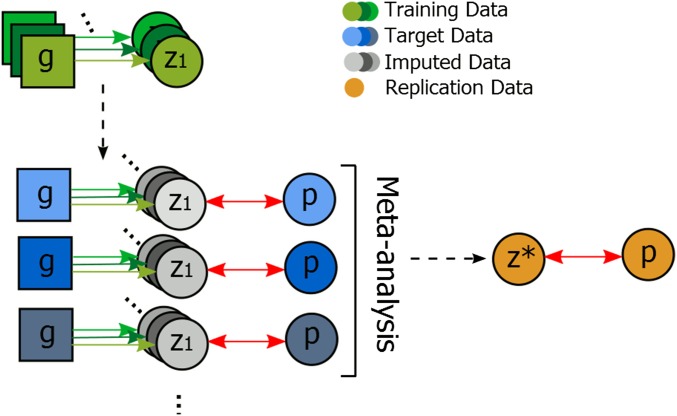
Schematic illustration of the employed methodology. Nodes indicate observable variables, with nodes of the same color indicating joint observations within individuals. Associations tested are indicated by red double-ended arrows. We combined information across various data sets. Using joint observations of genotypes and various intermediate phenotypes *z*, *i.e.*, tissue-specific observations of DNA methylation, given in a collection of training data sets we learn a collection of imputation functions (directed arrows). These were then applied to a series of GWAS data sets for Parkinson’s disease (p) to set up association tests with the individual imputed intermediate phenotypes and case–control status. Associations identified based on imputations were then replicated in a data set of DNA methylation measured in substantia nigra of cases with Parkinson’s disease and controls.

The general framework of the employed methodology, illustrated in Figure 1, builds on our previous work (Rowe *et al.* 2013) and is similar to transcriptome-wide imputation of gene expression (Gamazon *et al.* 2015). In brief, we aimed to assess potential association between DNAm in various relevant tissues, denoted by *z*, with some ultimate phenotype of interest *p*, in our case Parkinson’s disease. In the absence of a data set comprising joint observations of *z* and *p* we followed an imputation approach to constructing such a data set. Specifically, we made use of one data set of joint observations of genotypes **g** and intermediate phenotypes *z* to construct a predictor for the latter. This predictor was applied to a second data set of joint observations of genotypes and *p*, yielding a set of $\left(p,\hat{z}\right)$ pairs, with $\hat{z}$ being predicted observations of *z*. Using this data set we tested for association of *p* and $\hat{z}.$ We followed a linear mixed-model approach to imputation, as has been previously applied in this setting (Rowe *et al.* 2013).

We call this general approach a predicted intermediate trait association study (PITAS), that is, an imputation-based association study, and apply it using brain region-specific DNA methylation levels and Parkinson’s disease as the intermediate and ultimate phenotypes, respectively. Specifically we utilized case–control data from five GWAS of Parkinson’s disease (Table 1) in conjunction with a data set of DNAm from four brain tissues. In the following we describe the details of the imputation approach, its effect on statistical power, and the various data sets involved in this study.

**Table 1:** Summary of Parkinson’s disease case–control data sets used for imputation

	Individuals		
Study	*n* cases	*n* controls	SNPs
WTCCC	1,872	5,496	478,165
NGRC	2,000	1,986	314,434
phs000089	913	786	487,531
phs000126	860	873	302,619
phs000394	614	311	304,762
Total	6,259	9,452	

Study, dbGaP (http://www.ncbi.nlm.nih.gov/gap) study identifier or identifier used in main text; SNPs, number of SNPs available for imputation, *i.e.*, SNPs passing QC shared with the methylation data set.

### Statistical methods

Our imputation model, briefly summarized in the following, has been utilized in previous work (Rowe *et al.* 2013) and is similar to that used by Gamazon *et al.* (2015). Assume **g** is a vector of standardized genotypes for a set of *m* SNP markers, such that E[g]=0 and $\mathrm{var}\left(g_{i}\right)=1$ for all markers. Let $S_{z}$ be a sample containing observations of (g,z) pairs and $S_{p}$ an independent sample, from a related population, containing paired observations of **g** and *p*, where, as previously, indicated *z* denotes DNAm levels at a CpG site and *p* denotes the ultimate phenotype of interest, *i.e.*, in our case Parkinson’s disease status. We base imputation of *z* on the standard linear model for quantitative traits. Specifically we assume *z* arises from a linear combination of a subset of local marker effects and a further term capturing environmental and unmodeled genetic effects, so that for individual *i* we have

$$z_{i}=a^{T}x_{i}+b_{z}^{T}Hg_{i}+e_{i},\;\;e_{i}∼N\left(0,{\text{σ}}_{e}^{2}\right),$$

where $H\in {\left\{0,1\right\}}^{m_{z}\times m}$ is an incidence matrix selecting a subset of $m_{z}$ genetic markers local to the phenotype *z* and $b_{z}\;\in {\mathbb{R}}^{m_{z}}$ is a vector of marker effects. While several approaches for estimation of the marker effects exist, we use the empirical best linear unbiased predictor (eBLUP) (Rao 2003). The eBLUP arises by assuming a random-effects model such that $b_{z}∼N(0,\sigma_{g}^{2}{\text{m}}_{z}^{-1}I),$ obtaining restricted maximum-likelihood (REML) estimates ${\hat{\sigma }}_{g},\;{\hat{\sigma }}_{e}$of $\sigma_{g}$ and $\sigma_{e},$ and using these to estimate the effects as

$${\hat{b}}_{z}=\mathrm{argmax}\;P\left(b_{z}|{\hat{\sigma }}_{g},\;{\hat{\sigma }}_{e},\;S_{z}\right).$$

The estimated marker effects are then used to obtain an imputed intermediate phenotype ${\hat{z}}_{j}={\hat{b}}_{z}^{T}Hg_{j}$ for individual *j* in$\;S_{p}.$ Association of $\hat{z}$ and *p* can then proceed in the usual fashion based on a suitable statistical test.

### Specificity *P*-value

We define a specificity *P*-value as $P(t*<t^{z}),$ where t* and $t^{z}$ are test statistics for association of *p* with a random intermediate phenotype and z, respectively. We estimate a specificity *P*-value by generating 1000 alternative hypothetical intermediate phenotypes and comparing their association statistics with the statistic observed on the imputed intermediate phenotype. These generated intermediates were matched to the imputed intermediate phenotypes. Specifically, for the computation of a specificity *P* for imputed intermediate *z* we sampled marker effect vectors $b_{z}^{i}$ for i=1…1000 for the set of markers used for imputation of the corresponding intermediate phenotype. The effects were sampled according to the prior distribution $N\left(0,{\hat{\sigma }}_{g}^{2}m_{z}^{-1}I\right),$ where ${\hat{\sigma }}_{g}$ is the REML estimate of the genetic standard deviation for *z*. Generated intermediate phenotype *i* for individual *j* was then computed as $z_{j}^{i}=b_{z}^{i}{}^{T}Hg_{j}.$ Such alternative intermediates therefore represent samples from an absolute null, based on our prior assumptions on the distribution of marker effects. We also experimented with permutations of the marker effects estimated for *z* for generation of alternative intermediates, which yielded qualitatively similar results. As test statistics we used the *P*-values obtained from the test for association.

### Power of imputed intermediate trait association studies

The use of imputed intermediate phenotypes in lieu of their observations may adversely affect the statistical power to detect associations with the ultimate phenotype. The exact loss of statistical power will depend on many factors, including the relation of the intermediate and the ultimate phenotype and the estimator used. In general, we may analyze the statistical properties of the imputation methodology by adapting the framework of Dudbridge (2013), which itself represents a generalization to polygenic risk scores of the results by Daetwyler *et al.* (2008). To provide an indication of the effects that may be expected and an approximate guide we examine the effect of imputation in a simple scenario. Specifically, we consider the effect in the context of a continuous intermediate and ultimate phenotype within a fully linear model. Assuming standardized *z* and *p*, *i.e.*, E[p]=0, var(p)=1 and E[z]=0, var(z)=1, we adopt a linear model such that for individual *i*, $p_{i}$ and $z_{i}$ are related by

$$p_{i}=\beta^{0}+\;\beta z_{i}+\delta_{i},\;\;\delta_{i}∼N\left(0,\sigma_{\delta }^{2}\right),$$

where $\beta^{0}$ is an intercept and β∈(−1,1) is the effect of *z* on *p*. Note that for the standardized intermediate phenotype $\sigma_{g}^{2}$ equals the additive heritability $h^{2}.$ We furthermore restrict ourselves to the simpler least-squares estimator also used in previous analyses (Dudbridge 2013), rather than the eBLUP, which may be expected to yield better results. Formulas for alternative estimators and case–control data may be obtained trivially by substituting the expressions for variances and covariances derived below into the appropriate equations of Dudbridge (2013).

In general, the asymptotic noncentrality parameter of a $\chi^{2}$ test for association between some univariate *x* and *p* is

$$\lambda =\;\frac{N_{p}R^{2}}{1-R^{2}}\;\;\text{with}\;R^{2}=\frac{\mathrm{cov}{\left(x,p\right)}^{2}}{\mathrm{var}\left(x\right)\mathrm{var}\left(p\right)},$$

on 1 d.f., where $N_{p}$ is the number of joint observations. The power of the test at level *α* is given by

$$P_{\alpha }=1-\;\text{Φ}\left({\text{Φ}}^{-1}\left(1-\frac{\alpha }{2}\right)-\;\sqrt{\lambda }\right)+\;\text{Φ}\left({\text{Φ}}^{-1}\left(\frac{\alpha }{2}\right)-\sqrt{\lambda }\right).$$

For a study design with direct observations of the intermediate phenotype we may apply these formulas directly, taking x=z, in which case we have $R_{z}^{2}=\beta^{2}.$

Considering the use of imputations, we assume that the estimate follows $\hat{b}∼N\left(b,\sum_{\epsilon }\right),$ where the estimation noise covariance $\sum_{\epsilon }$ is estimator dependent, but will typically be a function of the training sample size $N_{z}$ and $\sigma_{g}.$ Under the additional reasonable assumption that the estimation error be independent of *p* we have $\mathrm{cov}\left(\hat{z},p\right)=\mathrm{cov}\left(z,p\right)-\mathrm{cov}\left(e,p\right)$ and $\mathrm{var}\left(\hat{z}\right)=\mathrm{var}\left(z\right)-\mathrm{var}(e)+\text{trace}\left(\sum_{\epsilon }\right).$ Hence a loss of power arises due to two factors, the failure to account for the environmental component, a function of the heritability of the intermediate phenotype, and the estimation error. Assuming genetic markers are not in strong linkage disequilibrium and genetic effects are small, a commonly made assumption (Dudbridge 2013), we may conservatively take $\sum_{\epsilon }\approx N_{z}^{-1}I\;$ so that $\mathrm{var}\left(\hat{z}\right)\approx h^{2}+mN_{z}^{-1\;}.$ Furthermore under the assumed model, we have $\mathrm{cov}\left(\hat{z},p\right)=\beta h^{2},$ so that $R_{\hat{z}}^{2}=\beta^{2}h^{2}/\left(1+m_{z}{\left(N_{z}h^{2}\right)}^{-1}\right),$ where we recall that $m_{z}$ is the number of markers used in imputation and $N_{z}$ is the sample size of the data set of observations of a specific intermediate phenotype.

### Data sources

We make use of a total of six publicly available data sets from the database of Genotypes and Phenotypes (dbGaP) that have been previously reported in the context of a GWAS for DNA methylation in brain tissue or Parkinson’s disease.

The first data set, which we refer to as the *methylation data*, was reported by Gibbs *et al.* (2010) and made available by The Division of Aging Biology and the Division of Geriatrics and Clinical Gerontology [National Institute on Aging (NIA)] through the NCBI Data Repositories (dbGaP accession phs000249.v1.p1, GEO series GSE15745). These data were obtained from postmortem samples of tissue from four regions of the human brain, specifically *cerebellum* (CRBL), *frontal cortex* (FCTX), *pons* (PONS), and *temporal cortex* (TCTX). The data comprise 150 individuals genotyped for 561,466 SNPs with the Illumina HumanHap550v3.0 platform, levels of DNAm from the four brain tissues for 27,578 DNAm sites quantified using the Illumina HumanMethylation27 Beadchip, and a list of potentially relevant covariates: sex, age, postmortem interval, assay plate, and study of enrollment.

The remaining data sets, referred to as the *Parkinson’s data*, comprise SNP genotypes, obtained with varying genotyping platforms, for individuals with Parkinson’s disease and unaffected control subjects. Three of the data sets were obtained through the NCBI Data Repository (dbGaP accessions phs000089.v3.p2, phs000126.v1.p1, and phs000394.v1.p1). In addition, we compiled a data set using Wellcome Trust Case Control Consortium (WTCCC) data sets obtained from the European Genome–phenome Archive (EGA). Specifically, the WTCCC data set comprises cases with Parkinson’s disease (EGA data set ID EGAD00000000057) and WTCCC2 controls from the 1958 British Birth Cohort and UK Blood Service Control Group (EGA data set IDs EGAD00000000022 and EGAD00000000024) genotyped using the Illumina platform. Finally, we used data for individuals with Parkinson’s disease and unaffected control subjects from the NeuroGenetics Research Consortium (NGRC), for which details regarding recruitment and quality control protocols have been reported previously (Hamza *et al.* 2010). Consistent quality control procedures as described below were applied across all five data sets, and information regarding individual data sets is summarized in Table 1.

### Quality control for genotype data

In all data sets genotyped samples were considered for downstream analysis if they were successfully typed for 95% of the SNPs assayed. SNP markers were included if they were typed in at least 95% of individuals within the data set, were in Hardy–Weinberg equilibrium at *P* ≥ 0.0001, and had a minor allele frequency ≥ 0.01. In the Parkinson’s data an additional quality control metric was applied and SNPs were included only if the difference in missing genotypes between cases and controls was not statistically significant (chi-square test; *P* > 0.00001). In both the methylation data and the Parkinson’s data the inbreeding coefficient *F* was estimated from the observed genotype frequencies of SNPs on the X chromosomes and it revealed each sample to be of recorded sex. Additionally, in the Parkinson’s data we excluded all individuals who shared a proportion >0.0625 of their genome identical by descent with any other individual in the same data set. In the methylation data, multidimensional scaling of the genotypic data and comparison with individuals from Yoruba in Ibadan (Nigeria), Japanese in Tokyo, Han Chinese in Beijing (China), and Utah residents with ancestry from northern and western Europe from the Centre d'Etude du Polymorphisme Humain collection (CEU) participating in the International HapMap Project (International HapMap Consortium 2003) showed the population sample to be homogeneous and of northern and western European descent. Where available we used self-reported ethnicity to exclude individuals of none White-European descent from the five Parkinson’s data sets. Due to the lack of this information in the WTCCC data set we assessed ancestry based only on the first two principal components of the individuals in combination with individuals from the HapMap reference population (International HapMap Consortium 2003). Similar examination of the first two principal components of individuals in the other data sets with individuals from the HapMap did not suggest the need for additional filtering based on genetic ancestry. Using principal component analysis across all data sets from the methylation and Parkinson’s data we confirm that there is no stratification present among the data sets (Supplemental Material, Figure S1).

### Quality control of DNAm data

Tissue samples from each of the four brain regions were included for analysis if ≥95% of the 27,578 DNAm sites assayed were detected above background noise levels at *P* ≤ 0.01, leaving sample sizes of 102, 109, 120, and 125 for the CRBL, FCTX, PONS and TCTX, respectively. Similarly, individual DNAm sites were considered for downstream analysis if they were detected above background noise levels at *P* ≤ 0.01 for ≥95% of the samples assayed. Additionally, DNAm probes listed in the Illumina HumanMethylation450 v.1.2 Manifest File (available at http://support.illumina.com) as containing a SNP within their probe sequence were removed. Recorded sex of each sample was checked against the average level of DNA methylation calculated from CpG sites located on the X chromosome. The methylation levels of each CpG site were rank transformed and adjusted for all the available covariates, which were sex, age, time of the sample extraction from death, study of the sample, and processing plate of the sample.

### Analyses: quality of imputation

To assess the quality of imputation we used fivefold cross-validation for 125 samples and 1826 DNAm sites measured in the temporal cortex, which were found to have nonzero *cis*-heritability (*P* < 0.05) based on SNPs located within 1 Mb of a specific DNAm site. Accuracy was measured as the correlation between the residual and the predicted phenotype.

### Association of imputed DNAm and Parkinson’s disease

Imputation in each of the Parkinson’s data sets was based on postquality control SNPs shared by the data set with the methylation data. Previous work indicates that a significant portion of variation in methylation is due to *cis* genetic effects (Gibbs *et al.* 2010; Quon *et al.* 2013) and we imputed DNAm level based on local genetic variation from markers within ±1 Mb surrounding the DNAm site, using REACTA (Cebamanos *et al.* 2014). Distances were computed based on B37 positions and information of the DNAm site location found in the HumanMethylation27_270596_v.1.2 manifest file. Using this approach, local regions contained between 2 and 787 SNPs (Figure S2). We restricted imputation to DNAm sites for which the local variation explained a significant proportion of the phenotypic variation (*P* < 0.05).

Within each data set, association of imputed DNAm and case/control status was tested by means of a logistic regression, for each DNAm site in each of the four regions. Within each data set we adjusted the analysis for available covariates, which for all data sets included gender, age, and the first two principal components obtained from the genotypes. Additionally, we adjusted for the source of the sample in the Parkinson’s data set phs000394. Where available, age was taken as age of onset for Parkinson’s disease cases.

An overall measure of association was obtained by combining results across the five data sets in a meta-analysis. As the data sets stem from disparate studies of varying design and varying implementation details, we opted to base the meta-analysis on a random-effects model. Specifically, we combined the regression coefficients obtained by logistic regression within each data set, using the Hedges–Vevea procedure (Schmidt *et al.* 2009). Finally, we used a Bonferroni correction to account for multiple comparisons and obtain adjusted significance thresholds across all imputed DNAm sites for each of the four brain regions.

For significant sites we also fitted a model including local genotypes. Specifically we fitted a genomic linear mixed model with a genomic relationship matrix computed on genotypes local to the CpG site and including predicted DNAm at the site as an additional fixed effect. We fitted individual models in each of the Parkinson’s data sets and combined the estimates of the effect of predicted DNAm, using the Hedges–Vevea procedure.

GWAS results are based on the same procedure as used for testing imputed methylation associations. Specifically, we also used logistic regression, including the same covariates, within each data set, with all SNPs available within each data set that passed quality control included in the individual GWAS. Results were combined across studies, using the Hedges–Vevea procedure, and genome-wide significance was based on a Bonferroni-adjusted threshold of 0.05.

### Reproduction in substantia nigra

DNA methylation was assayed using the HumanMethylation450 Beadchip in substantia nigra tissue samples of 40 cases and 44 controls obtained from the Parkinson’s UK Brain Bank and the Medical Research Council (MRC) Edinburgh Brain and Tissue Bank. We applied the same quality control procedures as described previously for DNAm data. With a significant age difference between the case and control groups (mean age 78.0 years for cases and 53.8 years for controls) we restricted the analysis to individuals >60 years of age (39 cases, mean age 77.8 years; 13 controls, mean age 78.4 years) to avoid confounding of age and disease status effects. After QC four of the sites identified through imputation were present in our data (cg10917602, cg09038914, cg08929103, and cg02301815). We tested for differential methylation between cases and controls at these sites by performing a logistic regression of methylation level on case–control status, while adjusting for the covariates age, gender, and postmortem interval.

### Data availability

The authors state that all data necessary for confirming the conclusions presented in the article are represented fully within the article.

## Results

### Quality of imputation

**Figure 2:**
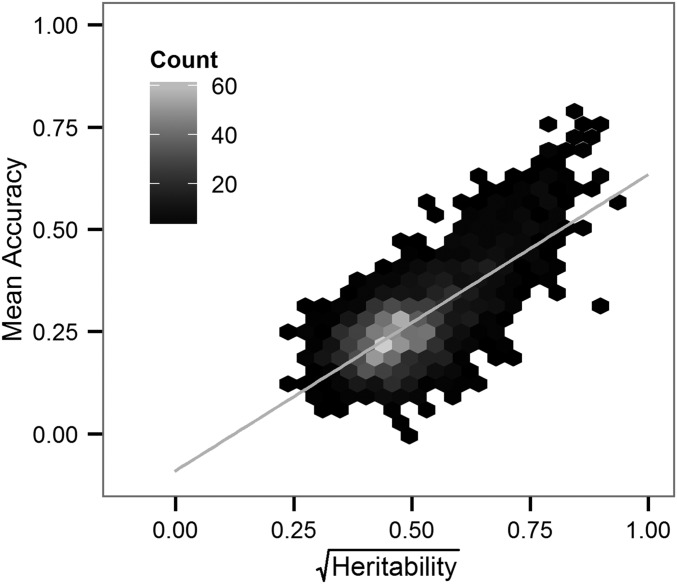
Cross-validation results for 1826 heritable DNAm sites measured in the TCTX. The mean accuracy across the five validation sets is plotted against the theoretical upper limit for the accuracy, the square root of the heritability. The line of best fit for the regression of the mean accuracy on the square root of the heritability is shown and is computed as y=0.73x−0.09 with *r*^2^ = 0.52.

Cross-validation demonstrated that accuracy of DNAm imputation from local SNPs was high. As seen in Figure 2, the mean accuracy across validation sets scaled, as expected, with the theoretical upper limit, which is the square root of the estimated trait *cis*-heritability. Overall the mean accuracy ranged from −0.0006 to 0.79 with an overall average of 0.27 (Figure S3). To assess the performance of imputation relative to this limit across all traits we normalized the mean accuracy across validation folds by dividing by the square root of the estimated trait *cis*-heritability. The normalized mean accuracies for the 1826 DNAm sites ranged between −0.0012 and 1.115 with a mean of 0.5409 and approximated a normal distribution (Shapiro–Wilk normality test; *P*-value for the deviation from normality = 0.2402). We verified that the resulting normalized mean accuracy below zero and greater than one, for one and three DNAm sites, respectively, could be attributed to sampling variation. Additionally, we found that the number of SNPs used to capture the regional effects did not explain a substantial proportion of the variance in the estimates of the normalized accuracy (*r*^2^ = 0.0580). Taken together these results suggest that for a majority of DNAm sites exhibiting significant *cis*-heritability, DNAm levels can be reliably imputed from SNP effects estimated in the same tissue.

### Comparison of statistical power

**Figure 3:**
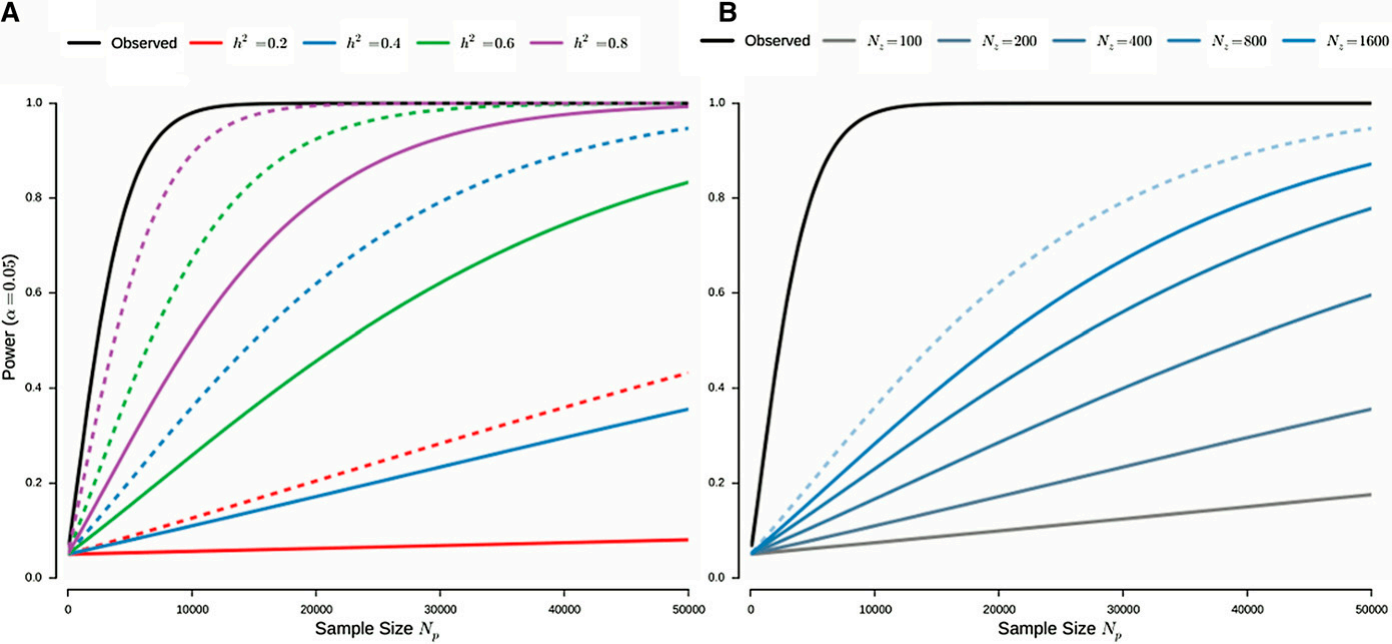
Effects of heritability and intermediate training sample size on statistical power. *Observed* denotes direct observations of the intermediate phenotype. (A) Achievable power as a function of the sample size of the target data for varying heritabilities of the intermediate phenotype. Dashed lines indicate the power for perfect predictions of the genetic component, providing an upper bound on the achievable power, and solid lines indicate the power with imputation based on $N_{z}=200$ observations of the intermediate phenotype. (B) The effect of $N_{z},$ the number of observations of intermediate phenotypes, for a fixed heritability of $h^{2}=0.4.$ In both A and B the effect size β is fixed to achieve a power of 0.8 when using 5000 direct observations of the intermediate phenotype, while $m_{z},$ the number of markers utilized for imputation, is 100.

We examined the loss of statistical power resulting from using imputed intermediate phenotypes as opposed to their observations. Using the derived expressions for the statistical power, we evaluate the power at level α as a function of sample sizes of the training and target data sets, $N_{z}$ and $N_{p};$ the number of genetic markers used for imputation, *m*; the effect of the standardized intermediate phenotype on the standardized ultimate phenotype, β; and the *cis*-heritability of the intermediate phenotype, $h^{2}.$ By *cis*-heritability, we are referring to the heritability captured by the local genotype that may not necessarily correspond to the total genomic heritability of the intermediate phenotype. Figure 3 illustrates the statistical power of imputation-based association testing for a realistic set of parameters. In particular, we evaluate the power as a function of the heritability of the intermediate phenotype (Figure 3A). Although imputation represents a severe penalty on statistical power, even if perfect predictions of the genetic component were to be obtained, for realistic sizes of imputation target data sets $N_{p},$ we may still expect to outperform the power achievable on data sets of direct observations, which may be expected to have sample sizes on the order of hundreds of individuals. We also considered the effect of the number of observations of intermediate phenotypes (Figure 3B). Specifically we evaluated the effect on statistical power across a range of sample sizes representative of currently available data sets, from the DNAm data sets used in this study ($N_{z}\approx 120$) to the whole blood gene expression data used by Gamazon *et al.* (2015) ($N_{z}\approx 900$). We observe that with modest increases of sample sizes for tissue-specific intermediate phenotypes, as are expected to become increasingly available through efforts like, for example, the GTex project (Lonsdale *et al.* 2013), we obtain significant gains in power. In particular, we note that even for sample sizes of 800, direct association testing on observations of the intermediate phenotype achieves, for the same effect size, only a power of 0.20 (at α=0.05).

### Association of predicted methylation with Parkinson’s disease

**Figure 4:**
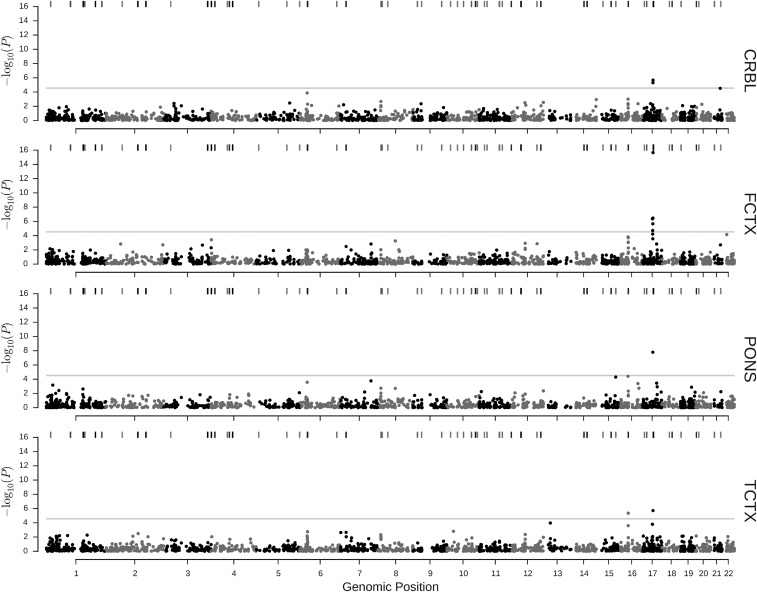
Heritable methylome-wide association *P*-values obtained by meta-analysis. The individual plots show *P*-values for association of imputed DNA methylation with Parkinson’s disease for four different brain tissues. In each tissue only heritable (*P* < 0.05) methylation sites were considered. The shaded horizontal line indicates significance within a tissue at a Bonferroni-corrected threshold of 0.05. Tick marks at the top of each plot indicate locations of Parkinson’s disease susceptibility loci previously identified by GWAS, obtained from the GWAS catalog (Welter *et al.* 2014) with shading indicating the *P*-value of the association (darker shading corresponds to lower *P*-values).

**Figure 5:**
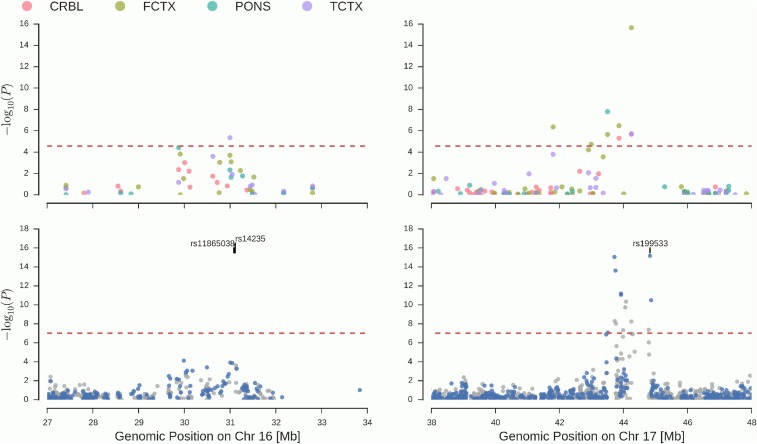
Regional comparison of GWAS and imputed DNA methylation association results. We show the *P*-values of association with Parkinson’s disease for SNPs (bottom) and imputed DNA methylation at heritable sites (*P* < 0.05) across four brain tissues (top) in regions containing significant predicted association of DNA methylation with Parkinson’s disease. Specifically, we show a region on chromosome 16 (left) containing cg10917602 (significant in FCTX and PONS) and a region on chromosome 17 near the previously reported association locus in *MAPT* containing five DNAm sites significant in at least one tissue (see Table 2). Dashed lines in the top plots indicate the most stringent imputed methylome-wide significance threshold of the four tissues and those in the bottom plots the genome-wide significance threshold for SNP associations. For GWAS, results for SNPs present in all five or only in a subset of studies are shown in blue and gray respectively, and additionally, locations of SNPs mentioned in the main text are indicated at the top of the plot.

Considering only methylation sites with *cis*-heritability (*P* < 0.05), we obtained predictions for 1793, 1717, 1635, and 1826 sites in CRBL, FCTX, PONS, and TCTX, respectively. Meta-analysis results across the different data sets for association are illustrated in Figure 4 and summarized in Table 2 (see Table S1 for full results and Figure S4 for forest plots of the effects). We found six methylation sites across two regions that showed significant association for at least one tissue after adjusting for multiple testing within tissue (adjusted *P* < 0.05). The relation of the GWAS and PITAS in regions surrounding these loci is illustrated in Figure 5. An isolated site, cg10917602 (TCTX), is located on chromosome 16. As can be seen in Figure 5, this site is not located near any SNPs showing significant association in our GWAS meta-analysis. However, it is <1 Mb from rs11865038 and rs14235, both reported previously (Pankratz *et al.* 2012; Nalls *et al.* 2014) to be associated with Parkinson’s disease susceptibility (at *P* = 4 × 10^−7^ and *P* = 2 × 10^−12^, respectively) and that represent the only variants on chromosome 16 contained in the GWAS catalog (Welter *et al.* 2014) in relation to Parkinson’s disease-associated traits. Neither of these SNPs is contained in any of the genotype data sets used in this study. Both of these SNPs are located at the edge of a linkage block in CEU HapMap individuals containing SNPs with elevated association with DNAm at cg10917602 (see Figure S5). However, the association of predicted DNAm at cg10917602 in TCTX with Parkinson’s disease status remained significant (*P* = 0.002) if a polygenic effect for the local genotypes was included in the model (Figure S6). All other sites to reach significance, cg02301815 (CRBL, FCTX, TCTX), cg07321605 (FCTX), cg08929103 (CRBL, FCTX), cg09038914 (FCTX), and cg14154330 (FCTX, PONS), are located in a 2.5-Mb region on chromosome 17. This coincides with a region containing a number of SNPs that were found to be significantly associated with case–control status, although cg07321605, the most telomeric DNAm site, is located ∼1.7 Mb upstream of the closest SNP reaching significance in the GWAS meta-analysis of our data, *i.e.*, rs11012. Furthermore, rs199533 (in *MAPT*), which is the strongest association (*P* = 6.7 × 10^−16^) within the region of significant SNPs in our GWAS meta-analysis, has been previously reported as being associated with Parkinson’s disease (Hamza *et al.* 2010), as have been various other SNPs in this region (Nalls *et al.* 2014).

**Table 2:** Summary of association results for imputed DNAm with Parkinson’s disease

Site	Chr.	BP	Tissue	*h*^2^	Association *P*	Specificity *P*
cg10917602	16	30,996,630	TCTX	0.14 (7.2 × 10^−4^)	4.5 × 10^−6^	0.003
cg07321605	17	41,804,527	FCTX	0.18 (4.8 × 10^−3^)	4.4 × 10^−7^	0.006
cg09038914	17	42,992,567	FCTX	0.14 (8.2 × 10^−3^)	1.9 × 10^−5^	0.007
cg14154330	17	43,503,401	FCTX	0.10 (3.4 × 10^−2^)	2.2 × 10^−6^	0.009
			PONS	0.18 (3.5 × 10^−4^)	1.6 × 10^−8^	0.001
cg08929103	17	43,860,355	FCTX	0.10 (1.9 × 10^−2^)	3.4 × 10^−7^	0.004
			CRBL	0.15 (3.0 × 10^−2^)	5.0 × 10^−6^	0.015
cg02301815	17	44,249,491	FCTX	0.11 (6.4 × 10^−3^)	2.2 × 10^−16^	<0.001
			CRBL	0.18 (1.5 × 10^−2^)	2.1 × 10^−6^	0.015
			TCTX	0.19 (5.4 × 10^−5^)	1.9 × 10^−6^	0.013

Chr., chromosome; BP, base pair position in genome build 37; *h*^2^, estimated regional additive DNAm heritability, with *P* against the null hypothesis of zero heritability given in parentheses; association *P*, *P*-values for association of DNAm and case–control status obtained by meta-analysis across all five Parkinson’s data sets; specificity *P*, probability of finding an alternative local intermediate phenotype of matched *h*^2^ with smaller association *P*, estimated based on meta-analysis of 1000 alternative intermediate phenotypes.

To assess whether identified associations were a consequence of a general enrichment of disease-associated genetic markers in the proximity of the known genetic association loci or specific to the DNAm phenotype, we followed a simulation approach. If the association is not specific to DNAm but a consequence of the general enrichment of genetic markers, we would expect alternative intermediate phenotypes, of equal heritability, to also show association with the ultimate phenotype. Hence, to assess the specificity of any observed association we estimated the probability of seeing an alternative simulated intermediate phenotype with greater association to the ultimate phenotype. We report this probability as the “specificity *P*-value” (see Table 2) and observe that all identified associations were found to be highly specific to the DNAm phenotype.

### Replication in substantia nigra tissue from cases with Parkinson’s disease and normal controls

We replicated the putative associations identified based on imputations using a data set of DNAm assayed in substantia nigra of 39 cases and 13 controls. Following quality control, we were able to test four of the six methylation sites identified previously, specifically cg10917602, cg09038914, cg08929103, and cg02301815. The cg10917602 site reproduced with *P* = 0.02 and a concordant direction of effect, with hypermethylation at the site associated with Parkinson’s disease susceptibility (change of one standard deviation in methylation level leads to a change in odds ratio of 0.27, 95% C.I. = [9.07 × 10^−2^, 0.81]), while cg02301815 reproduced with *P* = 0.03 but with a discordant effect direction (see Table S2 for full results).

## Discussion

We have demonstrated the viability of imputation-based association studies for tissue-specific DNA methylation in general. Specifically we have shown that a small discovery set consisting of samples taken from a relevant tissue is sufficient to predict with accuracy the genetic component of DNAm levels in the same tissue. These results are in contrast to the difficulties observed in predicting complex traits (for instance, disease risk) based on genotype information. This is, however, not unexpected due to two factors. First, the expected proximity, in terms of molecular interactions, of DNAm and the genotype leads to a reduction in biological noise. Second, the genetic variance is concentrated in *cis*-effects, which allows us to follow a regional approach that greatly reduces the number of model parameters, thus reducing the estimation error. These factors are not unique to DNA methylation but hold for other intermediate phenotypes as is demonstrated by results obtained in gene expression (Gamazon *et al.* 2015).

While we restrict imputation to the genetic component of the intermediate phenotype, it can be extended to include environmental components by utilizing estimated fixed effects during prediction, provided corresponding covariates have been observed in both estimation and the target data set. Including such effects may in general be expected to increase the power for detecting associations and may be of particular relevance for DNAm.

A natural question arising in the context of imputation, which previous applications failed to address, is the loss of statistical power. Statistical power to detect significant associations will in general be larger using direct observations of the phenotype compared to the same number of imputed observations. On the other hand, using imputation we may increase statistical power by augmenting any available data sets of relevant joint observations of intermediate and ultimate phenotype with additional data lacking observations of the former. In practice, a more relevant consideration is the allocation of resources in the design of a study. In particular, should investigators concentrate all resources on obtaining a joint data set, obtain a limited joint data set together with an extended GWAS data set, or concentrate purely on a GWAS data set for the ultimate phenotype? We have derived equations for the statistical power (see *Materials and* *Methods*) that allow for comparison of various designs within the first two options, allowing investigators to make informed decisions.

We studied associations between DNA methylation in four brain tissues and Parkinson’s disease and identified associations in two regions, only one of which is located near previously robustly identified disease-associated genetic variants. We are able to replicate the association in one of these regions based on direct measurements of DNAm in substantia nigra of cases with Parkinson’s disease and controls. However, because the replication size was modest, it would be desirable to perform further confirmatory replications in other well-powered cohorts. Despite this, it is worth highlighting that a standard GWAS meta-analysis applied to our data did not yield any significant associations near the identified CpG site on chromosome 16. This illustrates the potential of imputation approaches to identify novel associations. Considering the five sites upstream of the MAPT locus, failure to replicate for the three tested candidates could be a consequence of the lower power of the replication study. In particular, we may compare MAPT with other robustly reported Parkinson’s disease-associated regions, like, *e.g.*, the regions around SNCA and HLA on chromosomes 4 and 6, respectively (Hamza *et al.* 2010; Nalls *et al.* 2014), both of which contained significant associations in our GWAS meta-analysis. DNAm sites located in close proximity to these regions did not reach significance. Taken together with the specificity of the association with DNAm, as confirmed by the specificity *P*-values, this suggests that the observed associations at MAPT are not a consequence of an underlying enrichment of generic disease-associated markers in *cis* with the DNAm sites. Rather we may speculate about the role of DNAm in mediating the effects of genetic variation at the MAPT locus, noting that the distance between genetic marker and DNAm site is consistent with previous suggestions of DNAm as an intermediate of a genetic effect (Liu *et al.* 2013, 2014).

A challenge in the analysis of multiple phenotypes in observational data is the assignment of causality. While the chosen terminology of intermediate and ultimate phenotype might suggest a causal chain from genotype to intermediate to ultimate phenotype, it is important to note that we cannot distinguish between correlation and causation, and the direction thereof, in identified associations between intermediate and ultimate phenotypes. However, an important advantage of the imputation framework arises when applied to case–control data. Specifically, although we cannot discern causality, we may gain information concerning whether changes in cell phenotypes are an antecedent or a consequence of disease. This question, which cannot be addressed using a typical case–control design, is important for the construction of individualized disease risk predictors. Using an imputation methodology can help identify predictive associations, *i.e.*, differences in DNAm that predict disease risk. In particular, imputation may be used to establish whether associations identified in a standard case–control epigenome wide association study are predictive. To this end we may test for association of imputed rather than observed values at these sites. Provided these imputations are based on estimates obtained from a separate random sample of the population, they will be independent of disease status. Thus a significant association for imputed values provides evidence for the predictive nature of the changes found in the observed data. In our particular case we hypothesize that the association at cg10917602 is an antecedent of Parkinson’s disease, and a closer examination of methylation in this region as part of a prospective study could lead to the development of a marker for Parkinson’s disease risk and establish the causal nature of these changes.

## 
